# Astrocytes, a Promising Opportunity to Control the Progress of Parkinson’s Disease

**DOI:** 10.3390/biomedicines9101341

**Published:** 2021-09-28

**Authors:** Alberto Sanchez, Ingrid Morales, Clara Rodriguez-Sabate, Miguel Sole-Sabater, Manuel Rodriguez

**Affiliations:** 1 Laboratory of Neurobiology and Experimental Neurology, Department of Physiology, Faculty of Medicine, University of La Laguna, 38200 Tenerife, Spain; chova26@hotmail.com (A.S.); inmope@hotmail.com (I.M.); c.rsabate@hotmail.com (C.R.-S.); 2 Center for Networked Biomedical Research in Neurodegenerative Diseases (CIBERNED), 28031 Madrid, Spain; 3 Department of Psychiatry, Getafe University Hospital, 28905 Madrid, Spain; 4 Department of Neurology, La Candelaria University Hospital, 38010 Tenerife, Spain; solesabater@gmail.com

**Keywords:** Parkinson’s disease, astrocyte, dopamine neuron, cell treatment

## Abstract

At present, there is no efficient treatment to prevent the evolution of Parkinson’s disease (PD). PD is generated by the concurrent activity of multiple factors, which is a serious obstacle for the development of etio-pathogenic treatments. Astrocytes may act on most factors involved in PD and the promotion of their neuroprotection activity may be particularly suitable to prevent the onset and progression of this basal ganglia (BG) disorder. The main causes proposed for PD, the ability of astrocytes to control these causes, and the procedures that can be used to promote the neuroprotective action of astrocytes will be commented upon, here.

## 1. Introduction

Parkinson’s disease (PD) is produced by the confluence of multiple circumstances that, acting together, accelerate an aging-related degeneration of the nigrostriatal dopaminergic cells (DA-cells) and of other neuronal populations [1]. There is a growing interest in studying the involvement of astrocytes in the etiopathogenesis of PD, but their actual role remains unclear. Astrocytes may both protect and damage neurons, and it has been suggested that they prevent the onset but also accelerate the progression of PD. Astrocytes can modulate most of the multiple causes of PD, in some cases inhibiting their action but, in other cases, triggering their activation and facilitating their activity. As a result of this complex scenario, most of the treatments that are being evaluated for the prevention of PD do not include the action of astrocytes. It has been suggested that the first step of PD neurodegeneration may be produced in different brain areas or, even, outside the brain [2,3]. The PD brain presents structural changes in different neuronal types (e.g., noradrenergic neurons of the nucleus accumbens, dopaminergic neurons of the ventral tegmental area, GABAergic neurons of the striatum…), but the keystone of this disease is the degeneration of the dopaminergic nigrostriatal cells (DA-cells). The present study is focused on the actions of astrocytes on these neurons. The first part of the review presents a summary view of the main causes involved in the DA-cell degeneration, discussing how astrocytes can influence them either by preventing (slowing the PD progression) or promoting (accelerating the PD onset/progression) their activity. The second part of the review is focused on possible therapeutic strategies to facilitate the neuroprotective actions and to prevent the neurotoxic actions of astrocytes. Many of these strategies have been tested in animal models of PD but not in patients, and the final objective of this review is to encourage the development of clinical trials with therapeutic agents aimed at facilitating the neuroprotective actions of astrocytes in PD.

## 2. The Vulnerability of Dopaminergic Cells and Parkinson’s Disease

**Figure 1:**
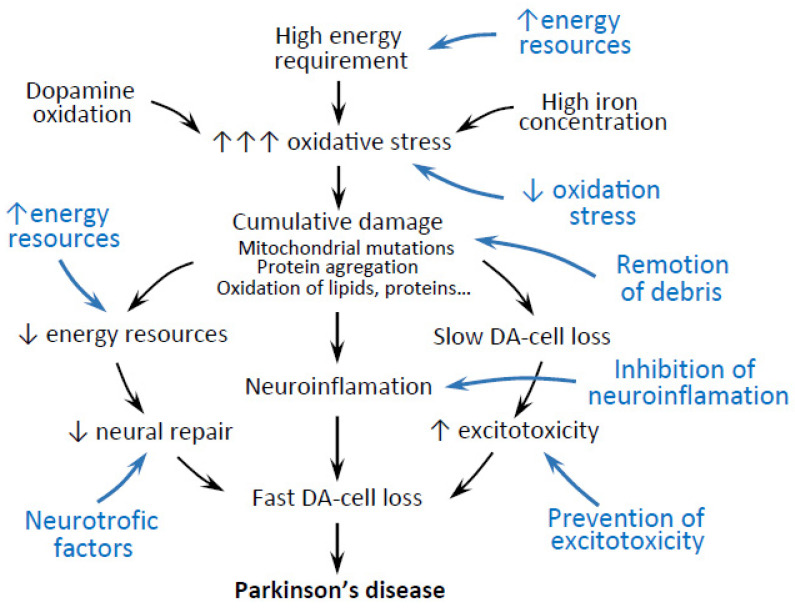
Etio-pathological factors (black) for DA-cell degeneration in PD and neuroprotective activities of astrocytes (blue).

DA-cells are particularly vulnerable to damage, as they suffer a progressive accumulative deterioration throughout life [4]. Human DA-cells have a long unmyelinated axon which repeatedly arborizes (total length exceeding 4 m) to produce up to 1 million synapses per cell [5]. The electrophysiological and neurochemical activity of the axon and synapses consume a large amount energy that needs to be continuously supplied by the hundreds of thousands of mitochondria distributed throughout the somata, axon, and synapses of DA-cells [6,7,8,9]. Between 0.5–2% of the oxygen consumed by mitochondria is transformed into reactive oxygen species (ROS; O_2_^−^, H_2_O_2_) which, together with the oxidative damage generated by the spontaneous oxidation of dopamine into dopamine quinone, disturb the mitochondrial activity, oxidize DNA, proteins and lipids which are necessary for DA-cell survival [7,8,10,11,12,13,14,15]. The accumulation of the mitochondrial damage (>70% of healthy people over 60 present massive mitochondrial mutations) produces a progressive energy imbalance that hinders cell repair and generates a slow loss of DA-cells (6–8% DA-cells each decade in the normal population). The regulatory mechanisms of the dopaminergic (DAergic) synapse prevent the clinical expression of the DA-cell loss, but in some people (2–3% of people over 60 years of age) the DA-cell loss accelerates (>10% cell loss/year), affecting more than 50–60% of DA-cells and inducing PD [4,16]. A number of structural and functional anomalies can be found in the DA-cell at this time, including mitophagy deterioration, proteasome malfunction, protein aggregation (e.g., α-synuclein in Lewy bodies), and neuroinflammation (e.g., with microglial activation) [17,18,19,20]. Many causes may be at the basis of the acceleration of the DA-cell degeneration, some of them might be induced by other cells of the BG (e.g., excitotoxicity induced by glutamatergic inputs), others by cells outside the blood brain barrier (BBB) (e.g., a transfer of peripheral inflammation to the brain), and others from the environment (e.g., slow intoxication with pesticides). Figure 1 (black) shows the main etio-pathological factors which have been involved in DA-cell degeneration. These deleterious factors normally act together, and the prevention of the evolution of PD should be oriented towards the simultaneous control of a substantial part of them. Astrocytes are able to perform this multiple approach (Figure 1 blue, Table 1).

## 3. Astrocytes Modulate the Vulnerability of DA-Cells in Parkinson’s Disease

Astrocytes are not a homogeneous cell group [88,89,90,91]. The main structural types of astrocytes are the fibrous astrocytes of the white matter, the protoplasmic astrocytes of the grey matter, and the specialized astrocytes of particular brain centres (e.g., Bergmann cells of the cerebellum) [92,93,94]. At the moment, the most relevant astrocytes in PD are the protoplasmic astrocytes, cells which express S100β, GLT-1, Aldh1L1, aquaporin-4, GFAP and µ-crystallin [93,95,96,97]. The expression of these proteins varies among the BG. For instance, the µ-crystallin level is higher in the protoplasmic astrocytes of the striatum than in the nigral astrocytes (which express more GFAP), and it is also higher in the ventral striatum (where it is found in 85% astrocytes) than in the dorsal striatum (30% astrocytes) [88]. The location of these proteins also varies inside the astrocyte structure. For instance, GFAP is normally found in the main processes of the astrocyte, whereas aquaporin-4 is mainly observed in the astrocyte processes that surround vessels to form the BBB [98]. Astrocytes also present marked changes with aging [99,100,101], increasing the expression of GFAP (which generates a flat morphology), accumulating iron (which disrupts the end-feeds and the BBB), and increasing the expression of cytokines (which induces a low-level chronic inflammation called neuro-inflammaging) [102].

Striatal astrocytes are massively coupled (−60 mV membrane potential) by means of gap junctions (the intracellular injections of dyes spread to >500 surrounding astrocytes in few minutes) [103]. Transmitters released by local neurons increase the intracellular Ca^2+^ of individual astrocytes, an effect that spreads to neighbouring astrocytes (Ca^2+^ wave) where it activates the release of glutamate and other transmitters (gliotransmission), thus conforming complex circuits with the medium-sized spiny neurons in the striatum [104,105]. Astrocytes express membrane transporters (e.g., glutamate and dopamine), membrane receptors (e.g., D2 dopamine receptor), and enzymes involved in the metabolization of neurotransmitters (e.g., glutamine synthetase for the metabolization of glutamate and monoamine oxidase for the metabolization of dopamine) [106].

Astrocytes respond to tissue damage (reactive astrocyte) by differentiating themselves into scar-forming astrocytes (generated from perivascular proliferating astrocytes not normally activated in PD), and hypertrophic astrocytes (generated from resident non-proliferating astrocytes that are normally activated in PD) [90]. Reactive astrocytes have toxic actions on synapses, neurons and oligodendrocytes (A1 astrocytes with complement 3 and MX dynamin-like DTPasa1 upregulation) or neuroprotective actions that activate synaptogenesis, inhibit apoptosis, and restore cell membranes (A2 astrocytes with upregulation of neurotrophics and expression of the S100A10 gen) [104,107,108,109,110]. Although the astrocyte reaction to damage adopts distinct molecular states in different diseases [111,112,113,114] and brain regions [114], and the A1–A2 binary classification may be considered as a simplification of the possible functional status of astrocytes [115], this classification is used here because it facilitates the presentation of data and because there is no commonly agreed alternative classification.

Human astrocytes have important differences to those of other mammals [116], showing intensive ramifications that cover a large tissue volume (a territory four times greater than that of rodent astrocytes) and modulate up to 2 million synapses [116,117]. Human astrocytes display a unique set of genes not found in other mammals, and some types of human astrocytes (e.g., interlaminar and varicose-projection astrocytes of the cortex) have not been found in other species [118,119]. These differences are at the basis of functional advantages which improve the cognitive functions of animals when they are implanted with human astrocytes [120].

Astrocytes perform both neuroprotective and neurotoxic activities, and the actual role of these cells in PD is dependent on the relative activation of these opposing functions [121]. DA-cells may not have efficient astrocytic support in the substantia nigra (SN), where astrocytes present a low cell-density and a low expression of neuroprotective factors in the SN, suggesting that their supporting activity is not particularly intensive in this center [121,122,123]. Animal studies have found a high expression of GFAP that decreases the activity of the glutamate transporters of nigral astrocytes, which facilitates the excitotoxic activity of the nigral glutamatergic inputs [124,125]. However, data reported in PD do not clarify this possibility, since although some studies found an increased GFAP level in the substantia nigra [126,127], other studies reported no differences [93,94,95]. Astrocytic support may also be low in the parkinsonian striatum, where astrocytes show a reduced expression of the neuroprotecting growth-inhibitory protein ROCK2 [128]. Some astrocytes could try to compensate for these deficiencies in PD [129] by increasing the expression of GBNMB (transmembrane glycoprotein with anti-inflammatory and anti-oxidant actions) [130,131] and CB2 (cannabinoid receptor which prevents neuronal degeneration by adjusting the cell metabolism) [132].

Different factors may obstruct the neuroprotective action of astrocytes. The beginning of PD may be facilitated by an age-related malfunction of astrocytes that reduces their number, increases their cellular volume, facilitates the overlap of their processes, and increases their GFAP content [102,133,134,135,136]. PD may also be facilitated by changes in the activity of different genes which increase the incidence of PD and are directly involved in the astrocyte biology [137,138,139,140,141,142,143,144,145,146,147,148,149]. This is the case of PARK7 (DJ-1 protein), which is involved in the glutamate uptake, mitochondrial function, oxidative stress, and inflammatory response of astrocytes [150,151,152,153,154]; PARK2 (Parkin), which is involved in the inflammatory response, neuroprotection, proliferation, and mitochondrial functions of astrocytes [149,155,156,157]; SNCA (α-synuclein), which is involved in glutamate uptake, neurotrophic activity, water transport, and endocytosis functions of astrocytes [158,159,160,161,162]; PINK1 (PTEN-induced putative kinase 1), which is involved in proliferation and mitochondrial function of astrocytes [139,163]; GBA (β-glucorecebrosidase), which is involved in autophagy, lysosome functions, and mitochondrial functions of astrocytes [164,165]; LRRK2 (leucine-rich repeat kinase 2), which is involved in autophagy and lysosome functions of astrocytes [166,167,168]; ATP13A2 (lysosomal type 5 ATPase), which is involved in the neurotrophic activity, inflammatory response, and lysosome functions of astrocytes [169]; and PLA2G6 (group VI Ca^2+^-independent phospholipase A_2_), which is involved in inflammatory response and calcium signaling functions of astrocytes [170,171]. Thus, the significance that the effects of these mutations have on the onset and progression of PD are probably linked to alterations in the physiological activity of astrocytes.

The progression of PD could also be facilitated by a malfunction of the supporting activity of astrocytes secondary to a previous neurodegeneration of DA-cells. The accumulation of α-synuclein is an example of this process. DA-cells in PD present intracellular inclusions of proteins (Lewy bodies) whose main component is α-synuclein aggregation. The low α-synuclein level normally found in astrocytes, is substantially increased in the PD brain and this increase correlates with the severity of the DA-cell loss [172]. Although astrocytes are resistant to protein accumulation and facilitate the removal of dopaminergic detritus [38], an excessive accumulation of α-synuclein compromises the A2 neuroprotective functions (e.g., after losing a part of their glutamate transporters), and activates the A1 neurotoxic phenotype of astrocytes [162].

Thus, astrocytes have a number of efficient mechanisms that may prevent or promote DA-cell degeneration [39,148], with a fine balance between both actions being critical for the onset and progression of PD [40,123]. As will be described in detail below, both neuroprotective and neurotoxic mechanisms can be used to develop new therapies to prevent the start (by promoting A2 activity) and progression (by inhibiting A1 activity) of PD.

## 4. Are Astrocytes Involved in the Clinical Expression of Parkinson’s Disease?

The influence of astrocytes on the physiology of DA-cells is often studied in experimental animals, but their actual role in the human brain and in the clinical expression of PD remains practically unexplored. This may be explained by the lack of methods to, in vivo, study the activity of astrocytes in the human brain. It is likely that not all patients present the same astrocytic deterioration, and that the clinical expression of the disease may depend on the degree of deterioration of the astrocytes of the striatum, medial forebrain bundle and SN. However, without having the appropriate techniques this possibility cannot be adequately evaluated.

## 5. Controlling Evolution PD with Astrocyte-Based Therapies

The control of A1 and A2 astrocyte functions is a promising field for the development of new PD therapies. Many particular astrocyte-based therapies that have proved useful in animal models of PD have not been tested in PD patients. The administration of astrocyte products (e.g., GDNF) have shown no conclusive results in PD but, in most studies, these products were administered directly and not using the endogenous astrocytes to release the neuroprotective agent. In addition, most studies administered particular astrocyte products, and approaches aimed at producing a widespread activation of the supporting activities of astrocytes are few and far between. Neuroprotective therapies based on astrocytic products are discussed below.

### 5.1. Astrocytes Provide Energy Resources to DA-Cells

Astrocytes are efficient glycolytic cells, supplying the tricarboxylic acid cycle of neurons with lactate (“lactate shuttle”) and accumulating glycogen reserves that can be rapidly hydrolyzed to produce glucose on demand [21]. These astrocyte abilities are necessary for DA-cells which, as commented above, are normally subjected to a particularly high metabolic pressure that requires a constant energy support. Available evidence shows that the energy resources in the PD brain are normally deficient [22,23,24], and that the facilitation of astrocyte glycolysis decreases the DA-cell vulnerability in animal models of PD [25]. The facilitation of astrocyte glycolysis could be useful to prevent PD progression, a possibility that could be tested by increasing the energy bioavailability with **ketogenic diets** [41] or with **intranasal insulin** [42].

### 5.2. Astrocytes Prevent Oxidative Stress in DA-Cells

As mentioned above, DA-cells generate large amounts of ROS and free radicals whose deleterious effects need to be continuously prevented and repaired. Astrocytes are particularly efficient in maintaining redox homeostasis, expressing different transporters (e.g., Cys-Glu antiporter which provides cystine for glutathione synthesis), exchangers (e.g., Glu-Asc exchanger which releases ascorbate for preventing DA oxidation), and enzymes (e.g., glutation peroxidase, glutation S-transferase, catalase and thioredoxin reductase that remove free radicals) that prevent the pro-oxidant action of H_2_O_2_, nitric oxide, peroxinitrites, and of the dopamine oxidative metabolism [26]. Astrocytes are the predominant source of glutathione, a tripeptide that protects cells from the oxidative action of superoxide radicals, hydroxyl radicals, peroxynitrites, and quinones, and which is selectively reduced (40%) in the SN of PD patients [27]. Glutation does not cross the BBB and its administration cannot prevent oxidative stress in the brain [28]. **N-acetyl cysteine**, an N-acetyl derivative of the naturally occurring amino acid L-cysteine, can be used to increase the synthesis of glutation and, therefore, to prevent the pro-oxidant action of H_2_O_2_, nitric oxide, peroxinitrites of DA-cells. There is evidence suggesting that its administration protects DAergic cells in animal models of PD and restores the DA-cell activity in PD patients [29,30,31]. The **N-acetylcysteine amide** (AD4) is another drug that crosses the BBB and facilitates the synthesis of glutation in the brain [32]. Some dipeptide precursors of glutathione (e.g., γ-**glutamylcysteine** and **cysteinylglycine**) can also reach the brain tissue, particularly when they are attached to nanoparticles formed from human serum albumin or when their chemical structure is modified to facilitate their liposolubility (e.g., **γ-glutamylcysteine ethyl ester**). Glutathione activation could also be induced with triterpenoids (e.g., **azadiradione** and **ursolic acid**) [33,34] or **salidroside** (Rhodiola rosea extract) [35]. The activation of the synthesis of glutathione in astrocytes may be useful to delay the evolution of PD, particularly if it is performed from the first stages of the illness and it is not interrupted.

Astrocytes express DA membrane transporters [36] and monoamine oxidases (MAO) [37], the latter of which decreases the oxidative stress produced by the DA degradation. MAO inhibitors (iMAO) (e.g., **selergiline**, **rasagiline**) which reduce oxidative stress in animals do not show a clear neuroprotection in PD [43]. This is probably because iMAOs produce antagonistic effects, directly decreasing the oxidative stress in DA-cells but indirectly reducing the dopamine metabolism in astrocytes (in such a way that the DA that is not metabolized in astrocytes produces free radicals in the extracellular medium or, after its uptake, in the DA-cells). iMAOs with a more selective action on DA-cells and few actions on astrocytes might probably be more useful for preventing the oxidative stress of DA-cells.

### 5.3. Astrocytes Prevent Excitotoxicity in DA-Cells

Astrocytes remove glutamate from the extracellular medium, thus preventing the excitotoxicity generated by the persistent activation of ionotropic glutamate receptors [44,45,46,47,48]. The PD brain presents excitotoxicity in both the striatum (where the DA decrease facilitates glutamate release, dendritic spiny loss, and the retrograde degeneration of DAergic axons) [44] and the SN (where the activation of the indirect pathway facilitates the glutamate release by the subthalamo-nigral projections) [49]. PD patients show a low basal glutamate uptake (50% reduction in the platelets of PD patients) [50] that can deteriorate with the DA decrease [51]. The pharmacological control of the glutamatergic synapses is challenging because glutamate is the transmitter of more than 70% of synapses all over the brain, and its modification can produce a number of side-effects. However, drugs that activate the glutamate transporter of astrocytes (e.g., **parawexin 1** -isolated from spider venom- and **ceftriaxone** -a β-lactam antibiotic-) or to blockade of the AMPA glutamate receptor of DA-cells (e.g., **talampanel**) could be useful to prevent excitotoxicity in PD [52]. The neuroprotective effect of **caffeine** in PD could be induced by a reduction of the release of glutamate in striatal astrocytes [53].

### 5.4. Astrocytes Prevent Neuroinflammation in PD

Astrocytes have become a major player in the neuroinflammation scenario, where microglia and the peripheral macrophages that infiltrate the brain had been practically the only important cells for many years [129]. The stimuli that activate neuroinflammation, the brain cells which detect these stimuli, and the moment when this process starts are still not well known in PD. Neuroinflammation may be triggered by brain stimuli (e.g., DA-cell debris that cannot be eliminated by A2 astrocytes) [38,54,55,56,57,61,129] or by a peripheral inflammation (e.g., induced by the gut microbiota) that cross the BBB [173,174]. The microglia may be the first cell that detect brain damage and triggers neuroinflammation. Microglial cells are continuously moving across the brain and, after detecting inflammatory stimuli, they change their M2 neuroprotective phenotype for a M1 neurotoxic phenotype which releases pro-inflammatory signals (e.g., IL-1α, TNFα, TGFα, NO, C1q) and converts A2 astrocytes into A1 astrocytes [175,176]. Protoplasmic astrocytes are distributed throughout the brain covering all the nervous tissue, and they may also be the first cells that detect damage and trigger neuroinflammation. In response to inflammatory stimuli, astrocytes change their A2 neuroprotective phenotype for a A1 neurotoxic phenotype which releases pro-inflammatory signals (e.g., orosomucoid-2, lipocalin, monocyte chemoattractant protein MCP-1/CCL2, IFN-γ inducible protein, pentraxin 3) and activates the production of M1 microglia [177]. Both A1 and M1 neurotoxic cells are probably involved in the PD neuroinflammation, where they remove neuronal detritus also inducing collateral damage to the DA-cells that are still alive. However, there is no clear evidence indicating which of these cells is activated first.

This scenario is at the basis of studies using anti-inflammatory drugs as a neuroprotection therapy in PD [129]. **Non-steroideal anti-inflammatory drugs** used to induce a non-selective blockade of neuroinflammation have not been shown to have consistent results [62]. Inconclusive results have also been reported after blocking the M1-microglia activity with **minocycline** [63]. **Doxycycline** is perhaps the most promising drug to prevent the M1 pro-inflammatory actions and protecting DA-cells in in vitro and in vivo animal models of PD [64,65]. This drug has a high tolerance in humans and should be tested in PD patients [66].

The mechanisms involved in the astrocyte–microglia interaction may be a suitable scenario for the selective control of the PD neuroinflammation. The stimulation of the DAergic receptors of astrocytes with D2-agonists (e.g., quinpirole, pramipexole) inhibits the pro-inflammatory activity of these cells [67], and this action is mediated by changes in the αB-crystallin expression [68,69] and the βarrestin2-mediated action of α-synuclein [70]. The M1 activation of the A2→A1 transition can be inhibited with both ***anti-TNFα medications*** (normally used for the treatment of inflammatory bowel disease and that has preliminary evidence for its neuroprotection in PD) [71] and **glucagon-like peptide-1 receptor (GLP1R) agonists** (used to control type 2 diabetes mellitus) [72]. The **NLY01** and **exendin-4** GLP1R agonists have proved useful for reducing DA-cell vulnerability in animal models of PD [73] and may also protect DA-cells in PD [74].

Astrocytes could also be involved in the modulation of the effect of peripheral inflammation on the brain. The BBB dysfunction found in PD patients [178,179] facilitates the access of products or cells generated by the peripheral inflammation to the brain [180,181,182]. The end-feet of astrocytes make contact with the brain vasculature surface, modulating the cerebral blood flow and the BBB permeability. The formation of tight junctions, the polarization of transporters [183] and the maintenance of the BBB activity [184,185] are modulated by astrocyte neurotrophins such as GDNF, VEGF, bFGF and ANG-1. The anti-oxidant activity of astrocytes and their release of neurotrophin prevent the deterioration of the BBB permeability, protecting the DA-cells from peripheral inflammation [180,186]. On the other hand, the astrocyte-mediated selective-opening of the BBB could help to facilitate the effectiveness of some types of PD treatment. This may be the case of monoclonal antibodies against α-synuclein or other toxic proteins involved in PD (**PD01A** and **PRX002/RG7935** affitopes are currently undergoing clinical trials).

### 5.5. Astrocytes Provide Neurotrophic Factors Which Are Necessary for DA-Cell Survival

Astrocytes release different neurotrophic factors (e.g., GDNF, BDNF, MANF, and *CDNF*) that protect DA-cells in animal PD models [75,76], but whose injection in the brain of PD patients has not produced the expected therapeutic effects [77,78,79,80]. Neurotrophins do not cross the BBB, and their direct administration in the brain tissue increases their concentration in the injection loci but not in the surrounding areas (they are rapidly metabolized by extracellular proteases). Thus, the brain region injected with neurotrophins presents an excessive dopaminergic re-innervation (that can generate dyskinesias and other undesirable side-effects), whereas the DA level in the surrounding areas remains low (thus generating the motor disorders of PD). In addition, the fast metabolization of neurotrophins limits the duration of their effects, which is an important limitation for treating chronic diseases with agents that need to be introduced into the BBB. The use neurotrophic factors could be significantly improved with new administration procedures that allow a sustained physiological increase of neurotrophins that may protect the whole brain and not only the local areas around the injecting cannulas. Astrocytes perform a precise control of their neurotrophins, releasing them at the exact time and place where their effects may be specific and balanced. The stimulation of this astrocytic activity could be a more effective way to control PD than the direct administration of neurotrophins. The injection of **lentiviral vectors** carrying the GDNF gene under the control of a GFAP promotor has proved to be useful to activate the astrocyte release of GDNF and to protect DA-cells in animals [81]. Drugs could be an alternative way to stimulate the astrocytic release of GDNF in humans. The administration of the grapefruit flavonoid **naringenin** is an example of how a drug which acts on astrocytes [82] can produce a moderate but persistent increase in the expression of GDNF and BDNF [83,84], thus protecting DA-cells in PD models [85,86]. The commonly-used spice cinnamon, and its metabolite sodium benzoate, are other examples, increasing the GFAP in the astrocytes of the SN and protecting DA-cells in animal models of PD [87].

### 5.6. The Global Activation of the Neuroprotective Functions of Astrocytes

**Figure 2:**
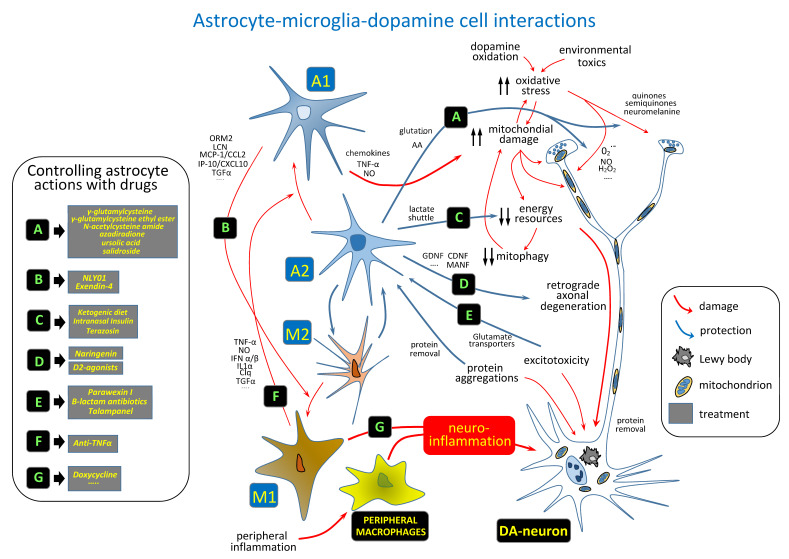
DA-cell interactions with astrocytes and microglia, and possible treatments to facilitate neuroprotection and to prevent neurodegeneration of DA-cells. ORM2: α-1-acid glycoprotein 2 precursor; LCN: lipocalin; MCP-1/CCL2: monocyte chemoattractant protein-1; IP10/CXCL10: interferon-gamma inducible protein; TGFα: transforming growth factor α; TNFα: tumor necrosis factor α; NO: nitric oxide; IFN α/β: alpha/beta interferon; IL1α: interleukin-1 α; CLq: component of the complement initiator C1 complex; AA: ascorbic acid; GDNF: glial cell line-derived neurotrophic factor; CDNF: cerebral dopamine neurotrophic factor; MANF: mesencephalic astrocyte-derived neurotrophic factor; Glu: glutamate.

The best therapeutic approach could be to induce a global activation of the A2 behavior of astrocytes preventing their evolution to the A1 phenotype. At present, there are no suitable procedures to perform a global control of the astrocyte activity, some therapeutic strategies may be useful. One possibility is to combine drugs that simulate the action of A2 astrocytes with drugs that inhibit the A2→A1 transition (see a summary of mechanisms and possible drugs in Figure 2). As mentioned above, the neuroprotective activities of astrocytes decrease with aging [99,100,101] and with the action of degenerating DA-cells [159,187,188], and another possibility could be to preserve astrocytes from these deleterious actions possibly by using genetic manipulations [189]. A further possibility is to replace the damaged astrocytes with new astrocytes obtained from iPS [190,191] or other sources [192]. The implant of human astrocytes has proved to be useful in animals (e.g., implanting human astrocytes in the brain of rodents; humanized mice).

The recent trials to facilitate the differentiation of nigral astrocytes to DA-cells goes in the opposite direction, as the objective of these trials is to increase the number of DA-cells has the collateral effect of reducing the local population of astrocytes. Astrocytes may be reprogramed by manipulating the genetic environment or by acting on specific pathways that facilitate their dedifferentiation to a pluripotent state that is later used to produce neurons [193]. The main methods for reprogramming astrocytes to neurons are the administration of transcription factors or microRNAs. Transcription factors are proteins that either up-regulate or down-regulate the transcription of specific genes, generally by interacting with the RNA polymerase’s transcription complex or by down-regulating others factors involved in stimulating transcription [194,195,196,197,198]. Currently, the reprograming of astrocytes into neurons may be performed with simpler procedures that overexpress a single transcription factor [193,199]. MicroRNAs are noncoding sequences of RNA (often of about 22 nucleotides) that may regulate gene expression at the posttranscriptional level, generally by selectively binding to a particular mRNA which produces the silencing of a specific RNA [200,201]. It has recently been reported that nigral astrocytes can be transformed into DA-cells by injecting an antisense oligonucleotide against the RNA-binding protein PTB in the SN [202]. This injection increases the number of DA-cells in the nigra and re-innervates the striatum with new DAergic synapses, thus recovering the dopamine level of 6OHDA lesioned animals to 65% of the control levels [202,203,204,205]. These studies were performed in young animals and it is currently unknown whether the astrocytes of aged animals may be also reprogramed to neurons. In addition, this method produces DA-cells but also other types of cells (e.g., GABA-neurons) whose activity could produce adverse side-effects, including the formation of teratoma [206,207]. Perhaps, the most prevalent problem generated by astrocyte reprograming in the medium term may be that the depletion of local astrocytes reduces the neuroprotective actions of these cells in the nigra, a fact that may increase DA-cell vulnerability and accelerate PD progression.

As commented above, the damage of mitochondria is critical for the progression of DA-cell degeneration. The massive damage of mitochondria of dopaminergic synapses (mainly induced by the over production of ROS) together with the fragmentation of the DA-cell axon (generated by the retrograde degeneration of DA-cells) prevent mitophagy in the PD brain. Mitophagy eliminates damaged mitochondria that produce low levels of chemical energy but high levels of ROS [7,8,10,13,14,208,209]. Thus, mitophagy decreases the progression of the DA-cell degeneration, and its obstruction may accelerate the clinical progression of PD. Astrocytes prevent this process by capturing and processing the damaged mitochondria of DA-cells (transmitophagy). Degenerating DA-cells store their mitochondria in saccular structures (spheroids) [38,57] that are later penetrated by astrocytic processes that transfer damaged mitochondria to astrocytes for their degradation [61]. Transmitophagy may prevent the release of damaged mitochondria into the extracellular medium where they activate neuroinflammation and accelerate the DA-cell degeneration [11,17,18,19]. On the other hand, there are preliminary data suggesting that the mitochondria of astrocytes may be transferred to neurons [58,59], a fact that, in the case of being produced in DA-cells, could compensate for the deleterious effects produced by their damaged mitochondria [60]. It has been suggested that astrocytes present mitochondrial dysfunctions in PD that facilitate the progression of the disease [149,210]. This mitochondrial dysfunction could originate in the mitochondria itself (e.g., induced by mutations of mitochondrial genes involved in PD) or it can affect the mitochondria indirectly (e.g., induced by α-synuclein or other proteins previously transferred by transautophagy of degenerating DA-cells) [38]. The damaged mitochondria found in astrocytes could also be those generated in DA-cells and which were later transferred to astrocytes by transmitophagy. In any case, the preservation of the protective role of astrocytes in the DA-cell mitochondria is probably critical for preventing the onset and progression of PD.

## 6. Final Comments

In summary, the supporting activity that astrocytes perform throughout life is necessary for the survival of neurons, and particularly of those neurons that, as occurs with DA-cells, have a high energy consumption and are submitted to high oxidative stress. The highly diverse neuroprotective and neurotoxic actions of astrocytes are a wide highway for the development of new therapeutic strategies in PD. Clinical researchers should pay more attention to astrocytes and to therapeutic approaches that, as described here, have shown promising results in animal models of PD. There are many possible therapeutic strategies that can be used to increase the neuroprotective action of astrocytes, some of them using drugs and other using cells or some of their components. Many of these strategies have already shown encouraging results in animal models and their effectiveness could begin to be tested in patients. The development of therapies to prevent the onset and progression of PD is complex and expensive. To be effective, many of the proposed therapies must be administered continuously and from the earliest stages of the disease. In addition, the demonstration of their effectiveness requires long-term studies and a prospective multicenter organization. The development of drugs for the symptomatic control of the clinical expression of PD is faster, easier, cheaper, and more profitable than the development of drugs for the etiopathogenic control of PD evolution. However, age-related neurodegenerative diseases are presently a growing “plague” with serious personal, family, work and social consequences, and the development of etiopathogenic therapies is a pressing social demand. Astrocytes should be considered for inclusion in these new developments.

## Figures and Tables

**Table 1:** Acting on the different stages of the PD evolution.

DA-Cell Requirements	Astrocyte Support	
high energy requirements	energy resource	[21,22,23,24,25]
high oxidative stress	antioxidant activity	[26,27,28,29,30,31,32,33,34,35,36,37]
cumulative damage	transautophagy	[21,22,23,24,25,26,27,28,29,30,31,32,33,34,35,36,37,38,39,40,41,42,43,44,45,46,47,48,49,50,51,52,53,54,55,56,57]
α-synuclein accumulation	α-synuclein remotion	[38]
mitochondrial damage	mitochondrial transfer	[58,59,60]
impaired mitophagy	transmitophagy	[61]
glutamatergic excitotoxicity	glutamate uptake	[44,45,46,47,48,49,50,51,52,53]
neuroinflammation	anti-inflammatory activity	[62,63,64,65,66,67,68,69,70,71,72,73,74]
need for trophic support	release of neurotrophic factors	[75,76,77,78,79,80,81,82,83,84,85,86,87]

## Data Availability

Not applicable.
